# A positive neighborhood walkability is associated with a higher magnitude of leisure walking in adults upon COVID-19 restrictions: a longitudinal cohort study

**DOI:** 10.1186/s12966-023-01512-3

**Published:** 2023-09-26

**Authors:** Marcia Spoelder, Merle C. A. Schoofs, Kevin Raaphorst, Jeroen Lakerveld, Alfred Wagtendonk, Yvonne A. W. Hartman, Erwin van der Krabben, Maria T. E. Hopman, Dick H. J. Thijssen, Lifelines Corona Research Initiative

**Affiliations:** 1 Department of Physiology, Radboud Institute for Health Sciences, Radboud University Medical Center, Philips Van Leydenlaan 15, Nijmegen, 6525 EX The Netherlands; 2 Present affiliation: Department of Primary and Community Care, Radboud Institute for Health Sciences, Radboud University Medical Center, Geert Grooteplein Noord 21, Nijmegen, 6525 EZ The Netherlands; 3 Department of Geography, Planning and Environment, Institute for Management Research, Radboud University, Nijmegen, The Netherlands; 4 Amsterdam UMC, Department of Epidemiology and Data Science, Vrije Universiteit Amsterdam, Amsterdam Public Health Research Institute, Boelelaan 1089a, Amsterdam, 1081HV The Netherlands; 5 Amsterdam Public Health, Health Behaviours and Chronic Diseases, Amsterdam, The Netherlands; 6 Upstream Team, Amsterdam University Medical Centers, Vrije Universiteit Amsterdam, Amsterdam, The Netherlands; 7 Research Institute for Sports and Exercise Sciences, Liverpool John Moores University, Liverpool, UK

**Keywords:** Walking, Built environment, Health, Physical activity, COVID-19

## Abstract

**Background:**

Previous cross-sectional and longitudinal observational studies revealed positive relationships between contextual built environment components and walking behavior. Due to severe restrictions during COVID-19 pandemic lockdowns, physical activity was primarily performed within the immediate living area. Using this unique opportunity, we evaluated whether built environment components were associated with the magnitude of change in walking activity in adults during COVID-19 restrictions.

**Methods:**

Data on self-reported demographic characteristics and walking behaviour were extracted from the prospective longitudinal Lifelines Cohort Study in the Netherlands of participants ≥ 18 years. For our analyses, we made use of the data acquired between 2014–2017 (*n* = 100,285). A fifth of the participants completed the questionnaires during COVID-19 restrictive policies in July 2021 (*n* = 20,806). Seven spatial components were calculated for a 500m and 1650m Euclidean buffer per postal code area in GIS: population density, retail and service destination density, land use mix, street connectivity, green space density, sidewalk density, and public transport stops. Additionally, the walkability index (WI) of these seven components was calculated. Using multivariable linear regression analyses, we analyzed the association between the WI (and separate components) and the change in leisure walking minutes/week. Included demographic variables were age, gender, BMI, education, net income, occupation status, household composition and the season in which the questionnaire was filled in.

**Results:**

The average leisure walking time strongly increased by 127 min/week upon COVID-19 restrictions. All seven spatial components of the WI were significantly associated with an increase in leisure walking time; a 10% higher score in the individual spatial component was associated with 5 to 8 more minutes of leisure walking/week. Green space density at the 500m Euclidean buffer and side-walk density at the 1650m Euclidean buffer were associated with the highest increase in leisure walking time/week. Subgroup analysis revealed that the built environment showed its strongest impact on leisure walking time in participants not engaging in leisure walking before the COVID-19 pandemic, compared to participants who already engaged in leisure walking before the COVID-19 pandemic.

**Conclusions:**

These results provide strong evidence that the built environment, corrected for individual-level characteristics, directly links to changes observed in leisure walking time during COVID-19 restrictions. Since this relation was strongest in those who did not engage in leisure walking before the COVID-19 pandemic, our results encourage new perspectives in health promotion and urban planning.

**Supplementary Information:**

The online version contains supplementary material available at 10.1186/s12966-023-01512-3.

## Introduction

Physical inactivity causes six to ten percent of the burden of major non-communicable diseases such as coronary heart disease, type 2 diabetes, and breast and colon cancers, and shortens life expectancy [1, 2]. Regular engagement in walking, a light form of physical activity, has been associated with reductions in all-cause mortality of 11% in healthy individuals [3] and up to 33% in patients with coronary heart disease [4]. Importantly, the largest decrease in mortality rates and the highest increase in general health is reached when physically inactive people become active [4–7]. Indeed, improvements in clinical outcomes have already been observed when comparing physically inactive individuals with those who engage as little as 10 min/day of brisk walking (i.e., 5.5 km/h) or 15–20 min/day of slower walking (3.2–4.0 km/h) [4]. Hence, the engagement in low-to-moderate-intensity walking is therefore a promising public health intervention target. Having access to a pleasant neighborhood living environment is especially important as it is here where the majority of walking activity is undertaken [8].

Individual and aggregated built environment components have been related to the engagement in physical activity, including walking [9–14]. The evidence is mostly derived from cross-sectional studies [15], but also numerous longitudinal studies and natural experiments do provide a positive relationship between the built environment and physical activity [16]. With regard to total physical activity, the individual built environment components with the strongest reported positive associations are population density, land use mix and access to public transportations as well as walking/cycling infrastructure [15, 16]. Studies addressing the relationship between the built environment and walking for transportation purposes have been more frequently investigated compared to leisure walking [15, 16]. Population density, street connectivity, accessibility and a new infrastructure show the strongest relationship with transport walking time [17, 18], whereas with regard to leisure walking, population density, accessibility to destinations and aggregate neighborhood typology have shown most often a positive relationship [15, 19–23].

Interactions and mobility were discouraged during the COVID-19 pandemic, limiting physical activity to the immediate living area of inhabitants. These extraordinary circumstances during COVID-19 policy restrictions represent a unique opportunity to study the relation between the built environment and (rapid changes in) walking behaviour. Previous studies have reported a drastic reduction in physical activity levels [24–27], with leisure walking (in close proximity to the immediate living environment) being one of the few options left to be physically active. Indeed, it has been shown that (leisure) walking strongly increased during COVID-19 lockdown restrictions [25]. 

The aim of the current study was to investigate whether and which of the included seven built environment components and the associated walkability index (WI), were related to the change in leisure walking time during COVID-19 lockdown restrictions. This investigation with a longitudinal character, may provide more direct evidence whether and which built environment characteristics are related to the changes in leisure walking time, since all inhabitants were affected. A secondary research question was to identify subject-related demographical factors that altered the relationship between the WI and the change in leisure walking time. For this purpose, we used a large longitudinal cohort of Dutch inhabitants who were examined pre-COVID-19 and during COVID-19 restrictions [28, 29]. Since no previous study investigated the association between the built environment and the change in leisure walking time due to COVID-19 restrictions specifically, we did not have a pre-defined hypothesis. However, since several previous studies showed a positive association between the built environment and walking, we hypothesized in general that a more favorable built environment, i.e. a higher WI score, is associated with more time spent on leisure walking. A better insight in the determining factors of walking under the COVID-19 restriction circumstances may encourage new perspectives in specific health promotion, urban planning, and inspire novel future strategies to design our outdoor living environment that facilitates walking.

## Methods

### Study design and participants

This study used data from the Lifelines Cohort Study. Lifelines is a multi-disciplinary prospective population-based cohort study examining in a unique three-generation design the health and health-related behaviours of persons living in the North of the Netherlands. The cohort employs a broad range of investigative procedures in assessing the biomedical, socio-demographic, behavioural, physical and psychological factors which contribute to the health and disease of the general population, with a special focus on multi-morbidity and complex genetics. Main characteristics of this longitudinal cohort of interest for this study are that people live in the same location/house for many years, have a uniform ethnicity (mainly Dutch) and show a diversity in social economic status. Further details of this cohort were described previously [29, 30]. The Lifelines protocol was approved by the UMCG Medical ethical committee under number 2007/152. For our analyses, we made use of the data of participants who are aged ≥ 18 years and completed the ‘second general assessment’ round (questionnaire completed between 2014–2017, of which the year and month of completing the questionnaire is known (to assess seasonality effects). Moreover, the data obtained in July 2021 of a specific COVID-19 subgroup of the general Lifelines cohort was used. This Lifelines COVID-19 cohort study was specifically established to investigate the health and societal impacts of COVID-19 and were recruited from the general Lifelines prospective cohort study [28]. A fifth of the participants who filled in their leisure walking time before the COVID-19 pandemic (*n* = 100,285), also completed the questionnaire during COVID-19 restrictive policies in July 2021 (*n* = 20,806), allowing us to investigate the difference in the reported leisure walking time before and during COVID-19. The prevalence of registered COVID-19 infections within the Netherlands largely fluctuated over time and in July 2021 again a peak of reported infections by the Municipal or Community Health Services was observed, fluctuating from 3,000–11,000 reported infections/day. The main restrictive measures active in July 2021 were the advice to work from home, closing of discotheques and clubs, restaurants and bars closed at 12pm with fixed seats, social distancing of 1.5m, wearing a mouth mask at airports and secondary schools. For a complete overview of the prevalence and restrictive measures within the Netherlands, we refer to the Dutch National Institute for Health and Environment [31].

### Dependent variable: minutes of walking

The primary outcome of this study was minutes of leisure walking per week. To assess this, the Dutch version of the Short Questionnaire to Assess Health-enhancing physical activity (SQUASH) was used [32]. This questionnaire divides the physical activity (PA) into four domains: 1) transportation to school or work (walking and biking), 2) light and heavy occupational PA, 3) light and heavy household PA, and 4) PA during leisure time. In the fourth domain, the included leisure-time activities are walking, cycling, gardening, odd jobs, and sports. Participants were asked to complete the duration and intensity of an individual’s typical weekly physical activities over the past month. The total minutes of leisure walking per week were calculated by multiplying the reported days per week times the minutes of walking per day. Unfortunately, the complete SQUASH questionnaire has only been provided to the Lifelines participants at the pre COVID-19 assessment. During COVID-19, in July 2021, only leisure walking and leisure biking was addressed within the specific domain of PA of leisure time, whereas this domain also includes gardening, odd jobs and sports. Hence, we were unable to calculate a valid change in the total PA measure based on the SQUASH questionnaire.

### Independent variables: walkability index (WI) and demographic characteristics

#### WI and the individual built environment components

Although many definitions of the walkability index concept exist, the general consensus is that the WI describes the extent to which the built environment stimulates walking behavior and that it can be used as a predicting factor for active mobility [33, 34]. The WI has been increasingly deployed and is shown to be useful and reliable to study the association between walking activity and the built environment [34–36]. In the Geoscience and Health Cohort Consortium (GECCO), a WI has been constructed for various exposure areas covering the whole of the Netherlands [34, 37–40]. The Dutch WI was based on the following seven spatial components: (1) population density, (2) retail and service destination density, (3) land use mix, (4) street connectivity, (5) green space density, (6) sidewalk density, and (7) public transport stops. These spatial components are described below:

- *Population density* at 2019 was defined as the number of residents per hectare, based on data from Statistics Netherlands of 100 × 100 m grids (Statistics Netherlands, CBS Statline [41, 42]).
- *Retail and service destination density* in 2017 was defined as the percentage of area devoted to retail, hospitality and catering industry, and social services (e.g., schools, medical services, religious buildings), based on land use data from Statistics Netherlands.
- *Land use mix* in 2017 was assessed using the entropy score (ranging from 0–1, with higher scores indicating a more heterogeneous land use mix): − 1*Σk(pk ∗ ln(pk))/ln(N), where p is the proportion of area devoted to a specific land use category (i.e., k), and N is the number of (aggregated or grouped) land use categories included. Data on the following land use categories were obtained from Statistics Netherlands: (1) residential areas, (2) commercial areas, (3) social-cultural services, (4) offices and public services, and (5) green space and recreation.
- *Street connectivity* at 2019 was defined as the number of road connections (including footpaths) per hectare of true intersections (i.e., three or more legs) on road segments that are accessible for pedestrians (e.g., excluding highways). The data on street connectivity were retrieved from the topographical TOP10 road intersection data in the Basic Topography Register System of The Netherlands’ Cadaster, Land Registry and Mapping Agency and from the data service of ESRI the Netherlands.
- *Green space density* in 2017 was defined as the percentage of area devoted to green space (i.e., parks, public gardens, forests, and cemeteries). The data on green space were retrieved from Statistics Netherlands.
- *Sidewalk density* was defined as the percentage of area devoted to sidewalks, and the relevant data were derived from the Key Register Large-scale Topography of the Netherlands Ministry of Infrastructure and Environment and from the data service of ESRI the Netherlands.
- *Public transport stops density* in 2018 are based on a point dataset with all public transport stops in the Netherlands (bus, ferry, metro, taxi, tram), but train stations excluded. The density in number of public transport stops is calculated and weighted with the number connecting lines per public transport stop. Public transport data were obtained from Geographic service of the University of Groningen (Geodienst Rijksuniversiteit Groningen, Groningen, the Netherlands).

In the present study, we derived the WI, and its (un)standardized components, from GIS data at 500m and 1650m Euclidean buffer zones of individual PC6. All seven WI components were produced as GIS raster layers with a 25 × 25 m raster cell resolution covering the Netherlands. The 500m and 1650m buffers were calculated for each raster cell using focal statistics, after which raster values have been spatially summarized per PC6 area with zonal statistics in GIS and exported in table format. Hence, the average value for each individual component has been calculated per PC6 area (part of zonal statistics) and these mean values (instead of the centroid value) were used. These PC6 GIS data was then linked to the Lifelines participants. To create the WI, the mean values of the zonal statistics were first standardized (i.e., converted into z-scores) and the linked standardized values were summed for the WI. Finally, the sum score was rescaled such that the WI ranged between 0 and 100, with higher scores representing higher walkability levels. No weights were applied to the components of the WI, since an equally weighted index seems to perform well in a Dutch context [34, 43, 44]. For detailed description and technical GIS operationalization of this WI, we refer to Wagtendonk and Lakerveld [40].

We had access to relatively fine-grained geographical areas of individual 6-digit postal codes (PC6). In the Netherlands, one PC6 consists of -on average- about twenty home addresses. The included participants of our whole investigate cohort of *n* = 100,285 at the pre-COVID-19 assessment, lived in 36,452 individual PC6 areas, out of the total 459,499 individual PC6 areas which have been identified within the Netherlands (determined in 2019), hence representing a coverage of 8%. The participants lived in 46 municipalities, out of the total 355 municipalities (determined in 2019), hence representing a coverage of 13%. Regarding the geographical sizes of our included PC6’s, it is important to stress that in the north of the Netherlands the sizes of the PC6 are quite different from each other due to the prevalence of large scale agricultural areas [45]. Within the inner cities administrative PC6 units are much smaller than those in regional or rural areas. The geographical size of the PC6 areas for the participants within the COVID-19 sub-cohort ranged from less than 1 km2 to more than 1000 km2: About 0.5% of the participants lived in a PC6 area of less than 1 km2, 45% in a PC6 area between 1 and 10 km2, 35% in a PC6 area between 10 and 100 km2, 14% in a PC6 area between 100 and 1000 km2, and 5% in a PC6 area between larger than 1000 km2.

#### Demographic characteristics

Demographic characteristics are potential confounding factors and were therefore included in the statistical analyses. The following characteristics, obtained in 2021 of the Lifelines COVID-19 cohort, were included: age, gender, body mass index (BMI), education level, net income, occupation status, household composition and seasonality. *Educational level* was categorized as ‘low education’ if they had no, lower vocational or low or middle secondary education as their highest finished education level. Respondents were classified as ‘middle education’ if they finished higher secondary education or middle vocational education and ‘high education’ for completing higher vocational education or university. For *net personal income*, respondents were asked: ‘what was your personal net income in the last month?’; with €500-step answer categories, with 12 categories in total. We redistributed these categories to three roughly equal-sized groups (low, medium, high). Individuals with a net income of €1500 or below were categorized as the ‘low income’ group. The ‘high income’ group consisted of people with a net income of €2500 or higher. *Occupation status* was included as a binary indicator (yes/no) when people worked either full-time, part-time or as freelance. For *household composition*, we included a variable with the binary indicators (yes/no) for being single (living alone) and another variable with the binary indicators (yes/no) for living at home with one or more children aged 18 years or below. Since participants were asked to report the duration and intensity of leisure walking time over the past month, the potential confounder of *seasonality* (spring, summer, autumn or winter), i.e. in which season the questionnaire was completed, was also taken into account because participants may spent more time on leisure walking during spring/summer compared to autumn/winter.

### Data analyses

Normally distributed continuous variables were presented as mean (± standard deviation; SD), and non-normally distributed data with the median [interquartile range; Q25-Q75]. For categorical data, the frequency with percentages were used to describe the data. All variables were visually inspected for normality as well as checked with the Shapiro–Wilk test. Pearson correlations were assessed between the WI and all standardized individual spatial components to examine the relationship between individual spatial components in the cohort. Potential differences in demographic characteristics at pre-COVID-19 (assessed between 2014–2017) between the general Lifelines Cohort and the Lifelines COVID-19 sub-cohort were tested using an independent T-test and Pearson’s χ2 test for continuous and categorical variables, respectively. We performed univariable and multivariable linear regression analyses to examine the relation between the changes in leisure walking minutes and built environment characteristics. The difference in the walking minutes (i.e. walking minutes in July 2021—walking minutes pre-COVID-19, i.e. difference score) was included as the dependent variable. The WI or the individual built environment components and the demographic variables were included as independent variables. Since we had no data on the change in total PA, the minutes of leisure walking at the pre-COVID-19 assessment was also included as an independent demographic variable, in order to adjust for regression to the mean [46]. Since our analyses showed that the pre-COVID-19 leisure walking time strongly influenced the size of the leisure walking difference score, we created a new variable in which we assigned the binary indicator (yes/no) to participants in whether they did or did not perform leisure walking at the pre-COVID-19 assessment, i.e. reported zero minutes of leisure walking. With the use of interaction terms, we investigated whether the incorporated demographic factor or the factor stating whether participants performed leisure walking pre-COVID-19 influenced the relationship between the WI and the COVID-19 related increase in leisure walking time. For the multivariable regression analyses, all independent variables were included within the statistical model (i.e. the enter model was used instead of a forward or backward selection). This was chosen to be able to compare the effect of individual built environment components on leisure walking time, since with the enter method all independent variables per regression analysis are included to calculate the size of effect on the dependent variable. Beta’s and 95% confidence intervals were provided for significant associations of the independent variable to the dependent variable. Since the individual PC areas largely differed in size (km2), we included a sensitivity analysis in which we divided the participants in three different PC area sizes (< 10km2, 10-100km2 and > 100km2). Besides the check for whether the residuals showed a normal distributions, also the other assumptions for multiple linear regression were checked. The analyses were performed in IBM SPSS Statistics (Version 26; IBMCorp, Armonk, New York, USA) and R version 3.6.3 and graphs were made in Graphpad Prism. *P*-values < 0.05 were considered statistically significant.

## Results

### Study population

In total, 23,863 participants reported their leisure walking time before and during the COVID-19 pandemic, of which 3,057 participants were excluded because they had moved, resulting in 20,806 participants who completed the questionnaire at both timepoints. At the pre-COVID-19 assessment, participants were aged 55 ± 11 years, the majority was female (60%) and the average BMI was 26 kg/m^2^. Education level, divided into low-medium–high, was roughly equally distributed, and 67% was employed. A small percentage (12%) lived alone, while 37% lived together with children under 18 years. The average leisure walking time per week was 139 ± 191 min with a median of 75 min. Of the participants, 12,213 (66%) indicated to perform leisure walking > 0 min. The average WI (range 0–100) was 22 and 32 at the 500m and 1650m Euclidian buffer GIS data, respectively. Demographic characteristics from the COVID-19 sub-cohort were different compared to the total Lifelines cohort (Table 1), with the COVID-19 sub-cohort being older, more often female, obtained a higher education, less often employed, lived less often with children under 18 years, and performed more leisure walking. The WI of the participants within the COVID-19 sub-cohort and the total Lifelines cohort was comparable but reached significance at the 1650m Euclidian buffer GIS data (Table 1). A graphical presentation of the number of participants of the COVID-19 sub-cohort per WI score in 2021 is presented in Suppl. Figure 1.

**Table 1:** Demographic characteristics and leisure walking minutes at pre-COVID-19 (assessed between 2014–2017) of the Lifelines COVID-19 sub-cohort and the total Lifelines cohort

**Variable**	**COVID-19 sub-cohort** ***N* = 20,806**	**Total Lifelines cohort** ***N* = 100,285**	***P* value**
**Age (years)**	55.1 ± 10.6	49.9 ± 12.7	*P* < 0.001
Median (min–max)	54 (20–89)	50 (19–96)	
**Gender**, female	12,485 (60.0%)	59,169 (59.0%)	*P* = 0.007
**BMI (kg/m**^**2**^**)**	26.1 ± 4.1	26.1 ± 4.3	*P* = 0.593
**Education**			
Low	5,180 (28.5%)	28,590 (29.2%)	*P* < 0.001
Intermediate	6,423 (35.3%)	36,244 (37.0%)	
High	6,586 (36.2%)	33,221 (33.9%)	
**Occupation status**			
Employed	12,365 (66.7%)	74,045 (74.2%)	*P* < 0.001
**Household composition**			
Living alone	2,124 (12.3%)	11,172 (12.5%)	*P* = 0.456
Having children < 18 yrs	6,537 (37.4%)	46,911 (50.5%)	*P* < 0.001
**# people who perform leisure walking**	12,213 (65.9%)	60,519 (60.8%)	*P* < 0.001
**Leisure walking minutes/week**			
Mean ± SD	139 ± 191	123 ± 185	*P* < 0.001
Median (min–max)	75 (0–2100)	60 (0–2310)	*P* < 0.001
IQR	180	180	
**WI score**			
** 500m Euclidian buffer** Mean ± SD	22.19 ± 13.49	22.11 ± 13.89	*P* = 0.456
** 1650m Euclidian buffer** Mean ± SD	32.41 ± 17.44	32.70 ± 18.30	*P* = 0.041

Categorical data were presented by count (percentage) and continuous data were presented as mean (± SD) and the median with min–max range. Independent T-tests and Pearson’s χ2 tests were used to assess potential differences between the COVID-19 sub-cohort and the total Lifelines cohort for continuous and categorical variables, respectively

*IQR* Inter-quartile range

### Pre-COVID-19: built environment components versus leisure walking time

Pre-COVID-19, the WI was significantly associated with leisure walking time within the total Lifelines cohort (Suppl. Table 1). A 10% higher WI was associated with 2.7 (95% CI; 1.8–3.6) and 1.2 (95% CI; 0.5–1.9) minutes increase in leisure walking per week at the 500m and 1650m Euclidian buffer GIS data, respectively (Suppl. Table 1). All included demographic variables, except education level, were significantly associated with leisure walking time (Suppl. Table 1).

### Change in leisure walking time during COVID-19 restrictions

**Fig. 1:**
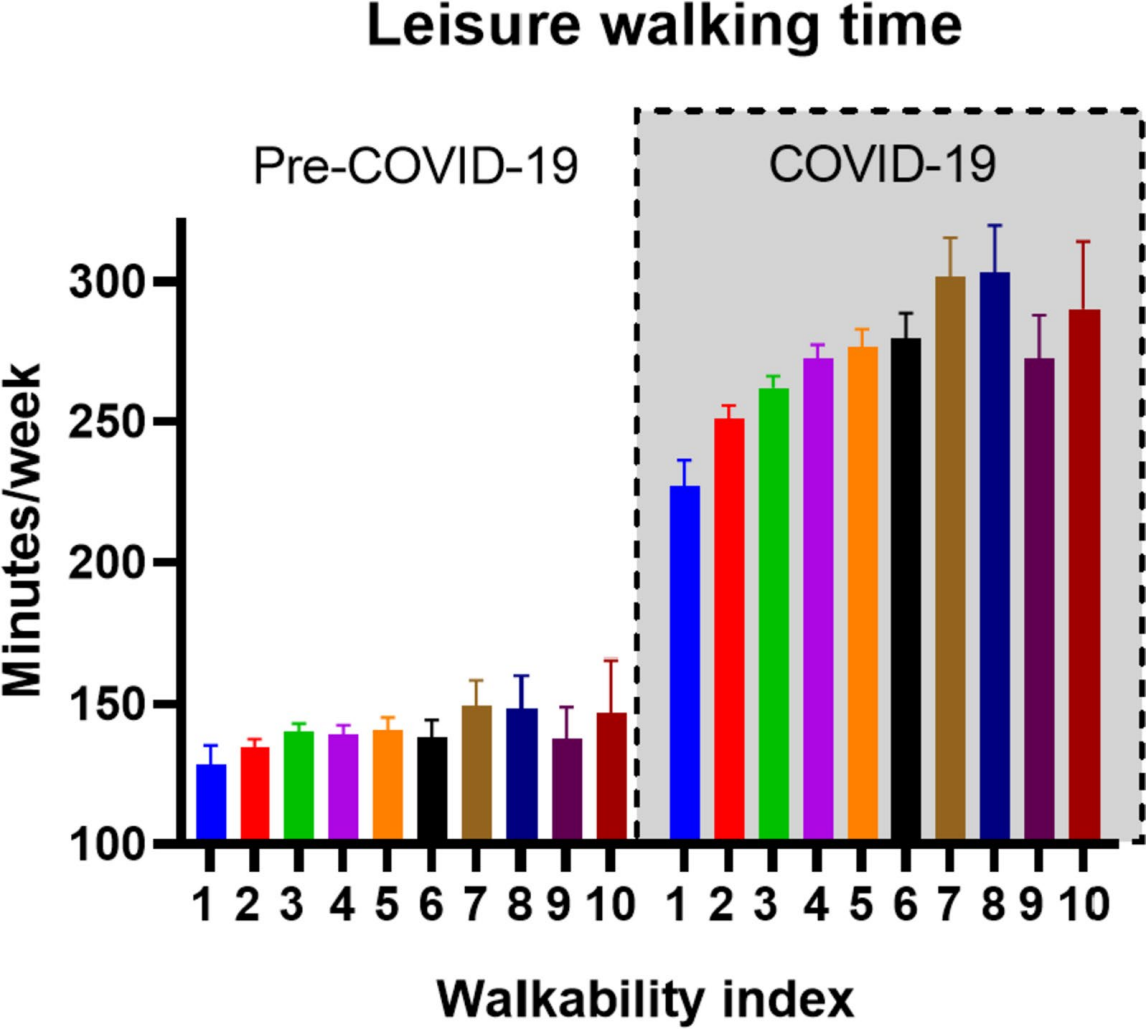
The COVID-19 related increase in leisure walking time was associated with the WI. The figure is based on the WI data with the GIS data of the 1650m Euclidian buffer. Number 1 to 10 indicate the WI in steps of ten percent

The number of participants who perform leisure walking significantly increased during COVID-19 lockdown restrictions, from 12,213 (65.9%) to 17,046 (82.1%). The average leisure walking time per week during COVID-19 lockdown restrictions increased with 127 ± 291 min/week (median:70; IQR:240), to 266 ± 285 min/week (median:180; IQR:300) (Fig. 1).

### Relation between the built environment and COVID-19 related changes in leisure walking

**Fig. 2:**
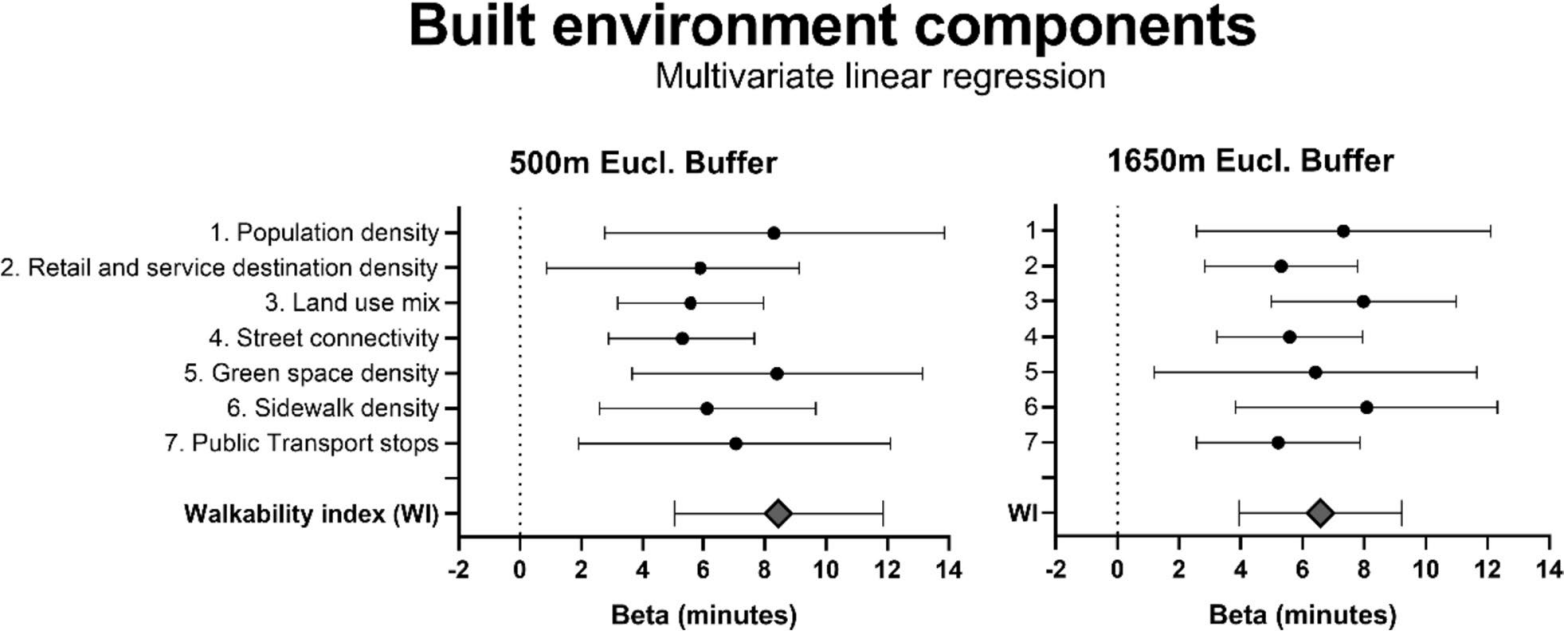
Forest plots depicting the effect estimates or Beta’s, i.e. increase in leisure walking minutes/week, of the individual built environment components and the WI with 95% confidence intervals. The shown Beta’s for the COVID-19 related increase in leisure walking time present the effect estimates with a 10% higher value of the individual spatial components and the WI. The multivariable model denotes fully-adjusted models including the WI or one individual built environment component and demographic factors (including: age, gender, BMI, education, net income, occupation status, household composition (both living with children < 18 yrs and/or living alone), seasonality and walking minutes during pre-COVID-19. Exact numbers and levels of significance are presented in Suppl. Table 2

The WI in the multivariable regression analyses was significantly associated with the change in leisure walking time. A 10% higher WI was associated with 8.5 (95% CI; 5.0–11.9) and 6.6 (95% CI; 3.9–9.2) more minutes of leisure walking/week for respectively the 500m and 1650m Euclidian buffer GIS data (Fig. 2). Hence, on average and based on the 1650m Euclidian buffer GIS data, participants living within a built environment with a WI score between 90–100, increased their leisure walking time during COVID-19 lockdown restrictions with 45 min/week, compared to participants living within a built environment with a WI score between 1–10 (Fig. 1).

Univariate and multivariable regression analyses for each individual built environment component indicated that all seven individual components were significantly associated with the increase in leisure walking minutes/week (Fig. 2; Suppl. Table 2). The level of green space- and side walk density showed to be associated with the largest increases in leisure walking time, at respectively the 500m and 1650m Euclidian buffer GIS data. A 10% higher green space density or side-walk density was associated with 8.4 (95% CI; 3.7–13.2) and 8.1 (95% CI; 3.8–12.3) more minutes of leisure walking per week, respectively. The relationships between the WI and the individual built environment components are shown in Suppl. Table 3 (500m Euclidean buffer GIS data) and Suppl. Table 4 (1650m Euclidean buffer GIS data). A sensitivity analysis in which the cohort was divided in three groups based on the size of the PC area (< 10km2, 10-100km2 and > 100km2), showed that the WI remained significantly associated with the increase in leisure walking time for each PC area size stratum (Suppl. Table 5). Interestingly, a 10% higher WI was associated with 22.3 and 13.3 more minutes of leisure walking for participants living PC areas > 100km, for respectively the 500m and 1650m Euclidian buffer GIS data. This is double or triple the effect estimates in leisure walking minute change compared to the smaller PC areas (Suppl. Table 5).

### The influence of demographic variables on the relationship between WI and the change in leisure walking time

The increase in leisure walking time from pre-COVID-19 to COVID-19 lockdown restrictions was 12.7 (95% CI; 0.1–25.3) minutes/week *higher* in participants who lived together versus participants who lived alone. The increase in leisure walking time was significantly 5.0 (95% CI; -6.1 to -3.9) minutes/week *lower* in participants with a 1.0 kg/m^2^ higher BMI value, 55.8 (95% CI; -68.3 to -43.2) minutes/week *lower* in participants who worked versus non-workers, and 13.4 (95% CI; 6.5–20.6) minutes/week *higher* in participants with a higher income level (Suppl. Table 6). Since, the income level was divided into three levels (low, medium, high), additional stratified analyses were performed and showed that individuals increased their leisure walking time with 118 ± 299, 131 ± 282 and 141 ± 281 min/week, with a low, medium, and high income, respectively (Suppl. Table 7). None of these demographic variables proved to significantly interact with the relationship between the WI and the change in leisure walking time in the multivariable regression analyses. The other included demographic variables, which entailed age, gender, education level, living with children < 18 yrs and the season at which the questionnaire was filled in, were not significantly associated with the change in leisure walking time.

We found a significant interaction-effect between WI and change in leisure walking time/week in individuals who already engaged in leisure time walking *versus* those who started leisure walking during COVID-19 lockdown restrictions (*p*-value; *p* = 0.005 at 500m- and *p* = 0.078 at 1650m Euclidian buffer). Stratified analyses on individuals who started walking for leisure during COVID-19 showed that a 10% higher WI was associated with an increase of 11.5 (95% CI; 5.6–17.4) and 9.0 (95% CI; 4.4–13.6) minutes/week for respectively the the 500m and 1650m Euclidian buffer GIS data, (Table 2), whilst for individuals who already walked for leisure at the pre-COVID-19 assessment, a 10% higher WI was associated with an increase of 5.4 (95% CI; 0.9–9.9) and 4.2 (95% CI; 0.7–7.6) minutes/week for respectively the 500m and 1650m Euclidian buffer GIS data (Table 2).

**Table 2:** Stratified analyses results for the multivariable linear regression for the relationship between the change in leisure walking time from pre-COVID-19 to COVID-19 restrictions and the WI. The effect estimates (Beta’s) for the COVID-19 related increase in leisure walking time were presented, denoting effect estimate with a 10% higher WI for the 500m and 1650m Euclidian buffer range and 95% confidence interval

**Stratum**	**Not walking** **during pre-COVID-19**	**Walking** **during pre-COVID-19**
**Number of participants**	6,328	12,213
**Increase in leisure walking minutes/week**		
** Mean ± SD**	208 ± 271	85 ± 292
** Median**	120	60
**Effect estimates when using the WI of 500m buffer GIS data**	11.5***(5.6;17.4)	5.4*(0.9;9.9)
**Effect estimates when using the WI of 1650m buffer GIS data**	9.0***(4.4;13.6)	4.2*(0.7;7.6)

Only fully-adjusted linear multivariable regression models were presented. Demographic factors included age, gender, BMI, education, net income, occupation status, household composition (both living with children < 18 yrs and/or living alone) and seasonality. significance level: ***: *P* < 0.001, **: *P* < 0.01, *: *P* < 0.05

## Discussion

We investigated the relation between built environment components and the change in leisure walking time during COVID-19 lockdown restrictions. First, we found that the walkability index (WI), and its individual built environment components, were significantly associated with leisure walking time during the pre-COVID-19 assessment. Second, during COVID-19 lockdown restrictions, we observed an average increase in leisure walking time of 127 min/week. Third, we found a strong and substantial influence of the built environment on the increase in leisure walking time. An additional 8.5 and 6.6 min of leisure walking per week was observed with a 10% higher WI at 500m and 1650m Euclidian buffer GIS data, respectively. Nearby green space- and side-walk density were associated with the largest increases in leisure walking time, when respectively the 500m and 1650m buffer size GIS data were used. Fourth, we observed that the association between the WI and the increase in leisure walking time was significant for participants living in both relatively small (< 10km2) and large (> 100km2) Postal Code areas. Finally, subgroup analysis revealed that the built environment showed its strongest impact on leisure walking time in participants not engaging in leisure walking before the COVID-19 pandemic, compared to participants who already engaged in leisure walking before the COVID-19 pandemic. This study is novel since we have measured the changes (pre-post) in walking behaviour and its relationship to multiple surrounding built environment components in a large sample size. In our study, the built environment remained unchanged, but people's relationship to it was influenced by COVID-19 lockdown restrictions. This is extraordinary because it is nearly impossible to experimentally manipulate attitudes towards the built environment at the scale needed to influence walkability.

Our study showed that the average leisure walking time, but also the number of participants who engaged in leisure walking, increased during COVID-19 lockdown restrictions. The effect size of the WI on leisure walking at pre-COVID-19 was 3-fold higher when exploring WI’s relation with the increase of leisure walking time during COVID-19 lockdown restrictions. This resulted in an additional increase of 45 min of leisure walking/week between those living in an area with the highest *versus* lowest 10% WI score. This observation suggests that the built environment contributes to both a priori engagement and the magnitude of additional leisure walking (during the COVID-19 lockdown). In line with earlier published findings [9, 34], the cross-sectional analyses of the leisure walking time taken at the pre-COVID-19 assessment showed a significant positive relation with the WI, indicating that participants spent more time walking for leisure when living in a favorable built environment. An important strength in our study design is that we were able to investigate the change in walking activity before and during COVID-19 restrictions, whilst keeping the built environment the same. Therefore, we were able to reduce the bias of residential self-selection often reported in cross-sectional observational studies [15, 17, 47–49]. When accounting for residential self-selection via a statistical- or (quasi) experimental design [15], a previous study indeed reported an attenuation of leisure walking time in high walkable neighborhoods after adjusting for reasons for moving to the new neighborhood [49], but still among the different individual built environment components, composite walkability indices often report consistent associations with PA [15]. Previous studies which were able to perform a pre-post assessment typically did so for one type of new built space (e.g. improved sidewalks, addition of a park), which often results in variable findings [15–17]. The advantage of using a composite approach, such as the WI, has been increasingly deployed and is shown to be useful and reliable to study the association between walking activity and the built environment [34, 36, 50–53]. Transportation walking, when defined as walking trips towards nearby shops and services, have more frequently published a positive correlation with walkability, compared to leisure walking [54]. With the use of the SQUASH questionnaire in our study, transportation walking was defined as the minutes of transport walking to school or work. Due to the low number of people reporting transport walking (15.3%) and a median score of 0 min in both the pre-COVID and COVID-19 restriction timepoints, we have not taken into account further analyses of this type of PA. Walking for leisure within the SQUASH questionnaire is not further specified in questions addressing the purpose of leisure walking. Hence, leisure walking can also be interpreted by individuals as a nice transport walk to the market in town to buy groceries, and does not necessarily needs to be related to green space or aesthetic conditions. Indeed, as reported in Fig. 2 and Supplementary Table 2, each component of the WI showed a significant association with leisure walking, including retail and service destination density.

We found that the relationship between the WI and the increase in leisure walking time was influenced by pre-COVID-19 leisure walking time. Specifically, a stronger relation between WI and the increase in leisure walking time was found in individuals who did not perform leisure walking before the COVID-19 pandemic. This is in line with an earlier observation in which the upgrades of parks (e.g. new or redesigned gymnasium, field improvements, walking paths, playgrounds) increased the number of first time park users [55]. Since the greatest health benefits are achieved when inactive people engage in moderate-intensity exercise, even if only for a few minutes a day [3], these insights highlight the potential importance of the built environment in supporting or promoting physically inactive individuals to start engaging in walking activities. This increase in leisure walking time is potentially highly relevant because before the COVID-19 pandemic, more than 50 percent of the Dutch inhabitants did not adhere to the recommended level of physical activity per week [56], and this percentage of not adherence seemed to build up during the COVID-19 pandemic [24, 26]. Since health-promoting activities are more often performed when they are in close proximity to people’s home [57], the promotion of walking may be a successful prevention strategy [9]. Furthermore, we found a higher increase in leisure walking time in men, participants with a higher net income (Suppl. Table 7) and in participants who lived together, whilst a lower increase in leisure walking time was found in those with a higher BMI and who worked. In contrast to these subject-related factors, we found no interaction between demographic factors *versus* the relation between the WI and the increase in leisure walking time. This suggests that the relation between the built environment and increase in leisure time walking is robust and is unlikely modified by demographic factors (e.g., socio-economic status) [58], and can be ascribed to the built environment characteristics.

The potential implication of our observations is that the immediate built environment may offer opportunities for environmental interventions. In this study, all seven built environment components were significantly associated with the COVID-19 related increase in leisure walking time, with green space and side wide density showing the largest effect sizes in leisure walking time. This is in line with an earlier reported positive relationship between walking and the quantity of parks, green strips and playgrounds [59]. Hence, modifying one or a few environmental attributes independent of other factors has to potential to encourage more walking activity. However, a detailed local analysis of the built environment in relation to its dominant demographic groups might be needed to translate the WI into concrete physical interventions. For example, more retail and service destinations within short distance (e.g. 1 km) likely increases walking time in sub-urban and rural areas [44], whilst much smaller effect sizes can be expected in urban areas that already have a high concentration of retail and service destinations [60, 61]. Previous investigations were conducted in participants primarily living in urban environments. In contrast, in our study, 67.8% of the participants lived in a rural area, defined as less than 1000 addresses/km2. The observation that the WI still played a significant role, might indicate that the importance of the built environment for leisure walking time applies for both urban and rural areas. Based on our sensitivity analyses in which we divided the participants in three groups based on the size of their PC area (< 10km2, 10-100km2 and > 100km2), we observed that the WI was significantly associated with the increase in leisure walking time for each PC area stratum (Suppl. Table S5). Interestingly, we observed that a 10% higher WI was associated with 22.3 and 13.3 more minutes of leisure walking for participants living PC areas > 100km, for respectively the 500m and 1650m Euclidian buffer GIS data. This is double or triple the effect estimates in leisure walking minute change compared to the smaller PC areas (Suppl. Table 5). This result is in line with the finding that leisure walking time increased the most for individuals that reported not to be engaged in leisure walking pre-COVID. It might be that these individuals reside mostly in the rural (and thus larger) PC areas. In fact, Lam and others previously indicated that the association between the WI and non-discretionary walking were higher in rural than urban areas [34]. Some studies are inconclusive or find a non-linear relationship between intersection density and walking behavior [36, 62]. This suggests that, as the WI is a composite score, local composites might contain trade-offs and differ depending on the spatial characteristics of a neighborhood. Comparable to earlier studies [36, 63], the largest effect size was seen when the GIS data of the smaller Euclidian buffer (500m) was used. This may indicate that the immediate environmental factors play an important role in influencing walking behaviour. This also stresses the importance of focusing on the direct living environment of inhabitants (e.g. within 1000m) instead of a further distance for the future spatial planning or adaptations in the built environment such as creating green space or certain facilities. Apart from modifying the built environment to encourage walking behavior, other health benefits may entail a higher social capital and improved mental health, which may have important implications for long-term health care savings [64–66].

Some limitations of our study must be considered. First, locational data were limited to the postal code level. PC6 areas are administrative zones that, while containing on average the same amount of addresses, differ greatly in absolute size depending on the level of urbanity. Consequently, PC6 areas should not be considered to be a constant spatial unit of representation for the direct living environment of both urban and rural participants. Especially for large, rural postal codes the use of zonal statistic values would yield questionable results. Since our data provide comparable outcomes when using the GIS data of the 500m vs 1650m Euclidian buffer, we assume our PC6 approach is robust to provide relevant insight. Related to the use of PC6, we were unable to apply a proper spatial autocorrelation [67]. A clustering based on the spatial area of PC6 is not possible with a multi-level analyses approach, since many of the included PC6 area in our analyses had a low number of participants/ PC6 area (66% of the PC6 entailed one participant). Moreover, the different sizes of the included PC6 areas in the north of the Netherlands due to the prevalence of large scale agricultural areas, also complicates spatial autocorrelation since the common approach using administrative boundaries (to define neighbors to include in Global Moran’s I spatial statistic) does not allow vastly varying sizes typical of administrative units [68]. Additionally, our large sample size also runs into the boundaries statistical programs can handle for spatial autocorrection [69]. We have considered and analyzed the relationship between the WI and the change in leisure walking time after clustering the PC6 areas into neighborhood codes, as done previously [70]. These results showed that the main conclusion of a strong associated between the WI and the change in leisure walking time remained, but due to the high loss of individual data, we did not decide to exchange the valuable individual data into neighborhood statistics to resolve spatial autocorrelation. A second limitation could be that the Dutch Walkability Index (WI) used in this manuscript, has not been specifically developed for leisure walking – but relates to total walking. An elaborate description regarding the theory-driven, evidence-informed approach in selecting components for the WI is provided by Lam and others (2022). The results in Fig. 2 and Supplementary Table 2 suggest that all separate components seem valuable. Therefore, we did not have a specific reason the exclude one of the individual components or alter weights. Moreover, it is relevant that the same approach of the Dutch WI is used to ease the comparison of study findings. Nevertheless, depending on the research question, it may be important to consider a difference in (the weights of) individual built environment components to include in a WI in future studies. A third limitation of our study is the use of the subjective SQUASH questionnaire to assess walking activity. In the SQUASH questionnaire, the purpose of the leisure walking activity was not assessed. Hence, it is unknown whether participants conducted their walking activity solely for leisure or whether they combined their walking activity with a purpose such as visiting a friend or doing groceries. Whether the enhanced engagement in walking during COVID-19 lockdown restrictions is sufficient to promote general health, or to compensate for the drop in physical activity, remains unclear [27], also in the Netherlands [24, 26].

## Conclusions

In conclusion, the COVID-19 related lockdown restriction provided a unique opportunity to understand the relation between the built environment and changes in leisure walking. Our results suggest that the immediate built environment WI -and especially its components green space- and side walk density-, is directly related to the increase in leisure walking behaviour. Interestingly, the WI showed its strongest impact on leisure walking time in participants not engaging in leisure time walking before the COVID-19 pandemic. These results provide strong evidence that the built environment, corrected for individual-level characteristics, directly links to leisure walking time and may encourage new perspectives in health promotion and urban planning.

### Supplementary Information

**Additional file 1:** **Supplementary Figure 1.** Graphical presentation of the frequency distribution of the WI of the included participants of the COVID-19 sub-cohort. **Supplementary Table 1.** Univariable and Multivariable linear regression results for the leisure walking time at the pre-COVID assessment (data acquired between 2014-2017). The effect estimates (Beta’s) are presented for the leisure walking time in minutes/week, denoting the effect estimates in both the 500m and 1650m Euclidian buffer and 95% confidence interval. **Supplementary Table 2.** Univariable and Multivariable linear regression results for the change in leisure walking time from pre-COVID-19 to COVID-19 restrictions for each individual spatial component of the WI. The effect estimates (Beta’s) for the COVID-19 related increase were presented, denoting effect estimates with a 10% higher WI or a 10% higher value in one of the individual spatial components in both the 500m and 1650m Euclidian buffer range and 95% confidence interval. **Supplementary Table 3.** Pearson correlations between the WI and all standardized individual spatial walkability components for the 500m Euclidean buffer zone^a^.**Supplementary Table 4.** Pearson correlations between the WI and all standardized individual spatial walkability components for the 1650m Euclidean buffer zone. **Supplementary Table 5.** Stratified analyses results per postal code area size for the relationship between the WI and the change in leisure walking time from pre-COVID-19 to COVID-19 restrictions. The average increase in leisure walking minutes and the effect estimates (Beta’s) for the COVID-19 related increase in leisure walking time are presented, denoting effect estimates with a 10% higher WI for the 500m and 1650m Euclidian buffer range and 95% confidence interval. **Supplementary Table 6.** Univariable and Multivariable linear regression results for the change in leisure walking time from pre-COVID-19 to COVID-19 restrictions. The effect estimates (Beta’s) for the change in leisure walking time are presented, denoting the effect estimates in both the 500m and 1650m Euclidian buffer and 95% confidence interval. **Supplementary Table 7.** Stratified analyses results of net income for the multivariable linear regression of the relationship between the change in leisure walking time from pre-COVID-19 to COVID-19 restrictions and the WI. The average increase in leisure walking minutes and the effect estimates (Beta’s) for the COVID-19 related increase are presented, denoting effect estimates with a 10% higher WI for the 500m and 1650m Euclidian buffer range and 95% confidence interval.

## Data Availability

Walkability data are available upon requests via GECCO website (www.gecco.nl). The 6-digit postal code environmental data are not publicly available at the resolution analyzed in this study due to regulations from the governing organization (Statistics Netherlands). 4-digit postal code data are however publicly available and can be downloaded free of charge via website: https://easy.dans. knaw.nl/ui/datasets/id/easy-dataset:103,498. Lifelines data will not be shared publicly due to individual privacy reasons. Access to the Lifelines data is organized according to a strict data access procedure. For all types of access, a research proposal must be submitted for evaluation by the Lifelines Research Office. The evaluation is performed to align the goals of the researchers with the goals of Lifelines (which are in turn aligned with the informed consent form signed by Lifelines participants). Further information on Lifelines data can be obtained by contacting the Lifelines Research Office (https://www.lifelines.nl).
