# Supplementary material for: A positive neighborhood walkability is associated with a higher magnitude of leisure walking in adults upon COVID-19 restrictions: a longitudinal cohort study
Source: Int J Behav Nutr Phys Act. 2023 Sep 26;20:116. doi: 10.1186/s12966-023-01512-3 (PMC10521432; doi:10.1186/s12966-023-01512-3)
Supplement: Supplementary file 1 — Additional file 1: Supplementary Figure 1. Graphical presentation of the frequency distribution of the WI of the included participants of the COVID-19 sub-cohort. Supplementary Table 1. Univariable and Multivariable linear regression results for the leisure walking time at the pre-COVID assessment (data acquired between 2014-2017). The effect estimates (Beta’s) are presented for the leisure walking time in minutes/week, denoting the effect estimates in both the 500m and 1650m Euclidian buffer and 95% confidence interval. Supplementary Table 2. Univariable and Multivariable linear regression results for the change in leisure walking time from pre-COVID-19 to COVID-19 restrictions for each individual spatial component of the WI. The effect estimates (Beta’s) for the COVID-19 related increase were presented, denoting effect estimates with a 10% higher WI or a 10% higher value in one of the individual spatial components in both the 500m and 1650m Euclidian buffer range and 95% confidence interval. Supplementary Table 3. Pearson correlations between the WI and all standardized individual spatial walkability components for the 500m Euclidean buffer zonea.Supplementary Table 4. Pearson correlations between the WI and all standardized individual spatial walkability components for the 1650m Euclidean buffer zone. Supplementary Table 5. Stratified analyses results per postal code area size for the relationship between the WI and the change in leisure walking time from pre-COVID-19 to COVID-19 restrictions. The average increase in leisure walking minutes and the effect estimates (Beta’s) for the COVID-19 related increase in leisure walking time are presented, denoting effect estimates with a 10% higher WI for the 500m and 1650m Euclidian buffer range and 95% confidence interval. Supplementary Table 6. Univariable and Multivariable linear regression results for the change in leisure walking time from pre-COVID-19 to COVID-19 restrictions. The effect estimates (Beta’s) for the c [file 12966_2023_1512_MOESM1_ESM.docx]

**Supplementary data**

**Supplementary Figure 1.**


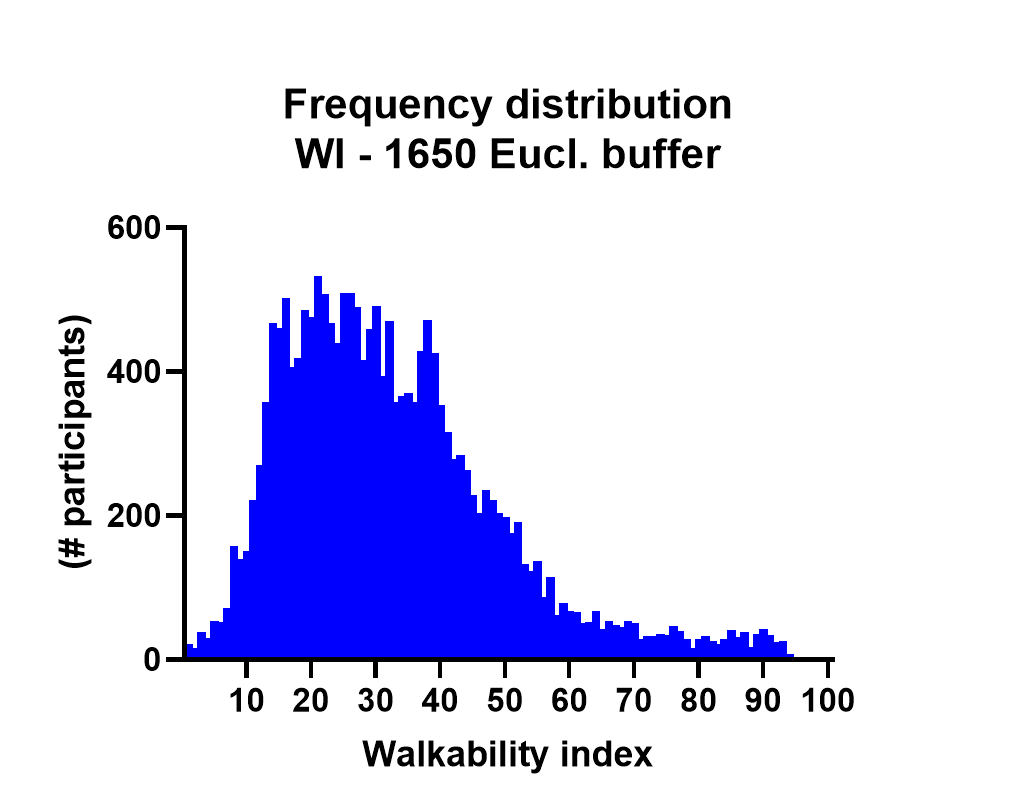


Supplementary Figure 1. Graphical presentation of the frequency distribution of the WI of the included participants of the COVID-19 sub-cohort.

**Supplementary table 1.**

Univariable and Multivariable linear regression results for the leisure walking time at the pre-COVID assessment (data acquired between 2014-2017). The effect estimates (Beta’s) are presented for the leisure walking time in minutes/week, denoting the effect estimates in both the 500m and 1650m Euclidian buffer and 95% confidence interval.

| **Independent variable:** | **Univariable** | **Multivariable**  **500m** | **Multivariable**  **1650m** |
| --- | --- | --- | --- |
| Walkability index (WI)  (range min-max: 0-10) | **500m: 2.75*****  (1.92;3.58)  **1650m: 1.32*****  (0.70;1.95) | **2.69*****  (1.76;3.62) | **1.22*****  (0.52;1.93) |
| Age | **0.47*****  (0.38;0.56) | **0.84*****  (0.72;0.96) | **0.83*****  (0.70;0.95) |
| Gender  *Ref: Female* | **-14.31*****  (-16.65;-11.98) | **-17.23*****  (-19.80;-14.66) | **-17.23*****  (-19.80;14.66) |
| BMI (kg/m^2^) | **-0.81*****  (-1.08;-0.54) | **-0.90*****  (-1.20;-0.60) | **-0.90*****  (-1.20;-0.60) |
| Education level | n.s. | n.s. | n.s. |
| Occupation status  *Ref: Not employed* | **-17.80*****  (-15.16;-20.44) | **-18.61*****  (-15.13;-22.09) | **-18.86*****  (-15.37;-22.34) |
| Household composition  *Ref: Living alone* | **10.58*****  (6.85;14.31) | **11.30*****  (6.92;15.67) | **10.53*****  (6.16;14.91) |
| Household composition  *Ref: Living without children* < 18 yrs | **-19.87*****  (-17.48;-22.26) | **-6.92*****  (-4.06;-9.79) | **-7.19*****  (-4.33;-10.06) |
| Seasonality | **-1.05***  (-2.05;-0.04) | **-1.47****  (-2.57;-0.37) | **-1.46***  (-2.56;-0.36) |
| Number of included participants | 88,703 to  99,597 | 84,009 | 84,009 |

The Univariable model denotes unadjusted models (only WI or the demographic variables) on leisure walking time. The Multivariable model denotes fully-adjusted models with the WI and the demographic variables confounders include age, gender, BMI, education, occupation status, household composition (both living with children < 18 yrs and/or living alone, seasonality. In case an individual component was not significantly associated with leisure walking time , it is reported as not significant (n.s.). P significance level: ***: P<0.001, **: P<0.01, *: P<0.05.

S**upplementary Table 2**. Univariable and Multivariable linear regression results for the change in leisure walking time from pre-COVID-19 to COVID-19 restrictions for each individual spatial component of the WI. The effect estimates (Beta’s) for the COVID-19 related increase were presented, denoting effect estimates with a 10% higher WI or a 10% higher value in one of the individual spatial components in both the 500m and 1650m Euclidian buffer range and 95% confidence interval.

| **Independent variable:** | **Univariable**  **500m** | **Univariable**  **1650m** | **Multivariable**  **500m** | **Multivariable**  **1650m** |
| --- | --- | --- | --- | --- |
| Walkability index (WI)  (range min-max: 0-10) | **6.38*****  (3.28;9.49) | **5.52*****  (3.12;7.92) | **8.45*****  (5.03;11.86) | **6.58*****  (3.94;9.21) |
| 1. Population density | **5.96***  (0.92;10.99) | **5.96****  (0.92;10.99) | **8.31****  (2.76;13.85) | **7.33****  (2.56;12.10) |
| 2. Retail and service destination density | **4.13***  (0.34;7.92) | **4.48*****  (2.21;6.76) | **5.90***  (0.87;9.12) | **5.31*****  (2.83;7.79) |
| 3. Land use mix | **3.96*****  (1.78;6.15) | **6.22*****  (3.49;8.96) | **5.57*****  (3.18;7.97) | **7.98*****  (4.98;10.98) |
| 4. Street connectivity | **3.99*****  (1.81;6.18) | **5.22*****  (3.06;7.38) | **5.28*****  (2.88;7.67) | **5.59*****  (3.22;7.95) |
| 5. Green space density | **6.58****  (2.22;10.93) | **5.00***  (0.21;9.79) | **8.40*****  (3.65;13.15) | **6.42***  (1.19;11.65) |
| 6. Sidewalk density | **4.32****  (1.10;7.54) | **7.26*****  (3.40;11.12) | **6.13*****  (2.58;9.67) | **8.08*****  (3.83;12.32) |
| 7. Public Transport stops | **6.06***  (1.35;10.78) | **4.16*****  (1.72;6.59) | **7.07****  (1.91;12.10) | **5.21*****  (2.56;7.86) |
| Number of included participants | 17,948 to 18,523 | 17,948 to 18,523 | 13,335 | 13,335 |

The univariable model denotes unadjusted models (only WI or its individual spatial components ) on the change leisure walking time (minutes). The multivariable model denotes fully-adjusted models with the WI or one individual spatial component and the demographic factors including: age, gender, BMI, education, net income, occupation status, household composition (both living with children < 18 yrs and/or living alone), seasonality and walking minutes during pre-COVID-19. P significance level: ***: P<0.001, **: P<0.01, *: P<0.05.

**Supplementary table 3.**

Pearson correlations between the WI and all standardized individual spatial walkability components for the 500m Euclidean buffer zone^a^

| Variable | 1 | 2 | 3 | 4 | 5 | 6 | 7 | 8 |
| --- | --- | --- | --- | --- | --- | --- | --- | --- |
| 1. Walkability index | 1.00 |  |  |  |  |  |  |  |
| 2. Population density | .77 | 1.00 |  |  |  |  |  |  |
| 3. Retail and service destination density | .72 | .46 | 1.00 |  |  |  |  |  |
| 4. Land use mix | .77 | .37 | .61 | 1.00 |  |  |  |  |
| 5. Street connectivity | .82 | .74 | .44 | .51 | 1.00 |  |  |  |
| 6. Green space density | .38 | .08 | n.s. | .36 | .26 | 1.00 |  |  |
| 7. Sidewalk density | .86 | .85 | .63 | .52 | .76 | .11 | 1.00 |  |
| 8. Public Transport stops | .72 | .59 | .54 | .39 | .44 | n.s. | .61 | 1.00 |

The walkability index (index range: 0–100) and the standardized components (z-scores) were derived from 500-m Euclidean buffer zones around the residential address of participants.

**Supplementary table 4.**

Pearson correlations between the WI and all standardized individual spatial walkability components for the 1650m Euclidean buffer zone

| Variable | 1 | 2 | 3 | 4 | 5 | 6 | 7 | 8 |
| --- | --- | --- | --- | --- | --- | --- | --- | --- |
| 1. Walkability index | 1.00 |  |  |  |  |  |  |  |
| 2. Population density | .88 | 1.00 |  |  |  |  |  |  |
| 3. Retail and service destination density | .92 | .83 | 1.00 |  |  |  |  |  |
| 4. Land use mix | .77 | .53 | .68 | 1.00 |  |  |  |  |
| 5. Street connectivity | .92 | .82 | .80 | .66 | 1.00 |  |  |  |
| 6. Green space density | .42 | .18 | .21 | .21 | .38 | 1.00 |  |  |
| 7. Sidewalk density | .92 | .91 | .88 | .62 | .90 | .24 | 1.00 |  |
| 8. Public Transport stops | .90 | .89 | .85 | .57 | .77 | .21 | .85 | 1.00 |

The WI (index range: 0–100) and the standardized components (z-scores) were derived from 1650-m Euclidean buffer zones around the residential address of participants.

**Supplementary Table 5**.

Stratified analyses results per postal code area size for the relationship between the WI and the change in leisure walking time from pre-COVID-19 to COVID-19 restrictions. The average increase in leisure walking minutes and the effect estimates (Beta’s) for the COVID-19 related increase in leisure walking time are presented, denoting effect estimates with a 10% higher WI for the 500m and 1650m Euclidian buffer range and 95% confidence interval.

| **Stratum** | **Small PC area**  **<10 km2** | **Medium PC area**  **10-100 km2** | **Large PC area**  **>100 km2** |
| --- | --- | --- | --- |
| **Number of participants** | 6,773 | 8,020 | 3,321 |
| **Increase in leisure walking minutes/week**  **Mean ± SD**  **Median** | 125 ± 291  75 | 131 ± 291  80 | 118 ± 289  60 |
| **WI of 500m Euclidian buffer GIS data** | 8.4**  (2.5;14.4) | 7.7*  (1.0;14.6) | 22.3**  (6.9;38.7) |
| **WI of 1650m Euclidian buffer GIS data** | 6.0**  (2.1;9.9) | 6.9**  (1.8;12.2) | 13.3*  (0.3;26.4) |

Only fully-adjusted linear multivariable regression models were presented. Demographic factors included age, gender, BMI, education, net income, occupation status, household composition (both living with children < 18 yrs and/or living alone), seasonality and leisure walking time at the pre-COVID-19 assessment. P significance level: ***: P<0.001, **: P<0.01, *: P<0.05.

**Supplementary table 6.**

Univariable and Multivariable linear regression results for the change in leisure walking time from pre-COVID-19 to COVID-19 restrictions. The effect estimates (Beta’s) for the change in leisure walking time are presented, denoting the effect estimates in both the 500m and 1650m Euclidian buffer and 95% confidence interval.

| **Independent variable:** | **Univariable** | **Multivariable**  **500m** | **Multivariable**  **1650m** |
| --- | --- | --- | --- |
| Walkability index (WI)  (range min-max: 0-10) | **500m: 6.38*****  (3.28;9.49)  **1650m:** **5.52*****  (3.11;7.92) | **8.45*****  (5.03;11.86) | **6.58*****  (3.94;9.21) |
| Age | **1.43*****  (1.03;1.83) | n.s. | n.s. |
| Gender  *Ref: Female* | **18.78*****  (10.24;27.33) | n.s. | n.s. |
| BMI (kg/m^2^) | **-3.92*****  (-4.92;-2.92) | **-5.02*****  (-6.12;-3.91) | **-4.98*****  (-6.09;-3.87) |
| Education level | n.s. | n.s. | n.s. |
| Net income  *Ref: low income* | **11.87*****  (5.69;18.05) | **13.84*****  (6.79;20.88) | **13.57*****  (6.52;20.62) |
| Occupation status  *Ref: Not employed* | **-35.70*****  (-44.06;-27.34) | **-55.69*****  (-68.22;-43.16) | **-55.76*****  (-68.27;-43.22) |
| Household composition  *Ref: Living alone* | n.s. | **12.90***  (0.30;25.50) | **12.70***  (0.11;25.29) |
| Household composition  *Ref: Living without children* < 18 yrs | **-14.50***  (-2.23;-26.77) | n.s. | n.s. |
| Seasonality | **3.94***  (0.34;7.54) | n.s. | n.s. |
| Leisure walking minutes pre-COVID-19 assessment | **-0.55*****  (-0.57;-0.53) | **-0.56*****  (-0.58;-0.53) | **-0.56*****  (-0.58;-0.53) |
| Number of included participants | 13,335 to 18,523 | 13,335 | 13,335 |

The Univariable model denotes unadjusted models (only WI or the demographic variables) on the change in leisure walking time (minutes). The Multivariable model denotes fully-adjusted models with the WI and the demographic variables confounders include, age, gender, BMI, education, net income, occupation status, household composition (both living with children < 18 yrs and/or living alone, seasonality and the walking minutes during pre-COVID-19. In case an individual component was not significantly associated with leisure walking time , it is reported as not significant (n.s.). P significance level: ***: P<0.001, **: P<0.01, *: P<0.05).

**Supplementary Table 7**.

Stratified analyses results of net income for the multivariable linear regression of the relationship between the change in leisure walking time from pre-COVID-19 to COVID-19 restrictions and the WI. The average increase in leisure walking minutes and the effect estimates (Beta’s) for the COVID-19 related increase are presented, denoting effect estimates with a 10% higher WI for the 500m and 1650m Euclidian buffer range and 95% confidence interval.

| **Stratum** | **Low income**  **<€1500** | **Medium income**  **€1500-2500** | **High income**  **>€2500** |
| --- | --- | --- | --- |
| **Number of participants** | 5,533 | 6,131 | 3,851 |
| **Increase in leisure walking minutes/week**  **Mean ± SD**  **Median** | 118 ± 299  60 | 131 ± 282  80 | 141 ± 281  90 |
| **Effect estimates when using the WI of 500m Euclidian buffer GIS data** | 9.1*  (3.3;15.0) | 8.3**  (2.8;13.7) | 7.5*  (0.9;14.2) |
| **Effect estimates when using the WI of 1650m Euclidian buffer GIS data** | 6.3**  (1.6;10.9) | 7.3***  (3.1;11.5) | 6.0*  (1.1;11.0) |

Only fully-adjusted linear multivariable regression models were presented. Demographic factors included age, gender, BMI, education, net income, occupation status, household composition (both living with children < 18 yrs and/or living alone), seasonality and leisure walking time at the pre-COVID-19 assessment. P significance level: ***: P<0.001, **: P<0.01, *: P<0.05.
